# Supplementary material for: Development and Validation of Nomograms for Malignancy Prediction in Soft Tissue Tumors Using Magnetic Resonance Imaging Measurements
Source: Sci Rep. 2019 Mar 20;9:4897. doi: 10.1038/s41598-019-41230-0 (PMC6427044; doi:10.1038/s41598-019-41230-0)
Supplement: Supplementary file 1 — Supplementary material [file 41598_2019_41230_MOESM1_ESM.docx]

**Development and Validation of Nomograms for Malignancy Prediction in Soft Tissue Tumors Using Magnetic Resonance Imaging Measurements**

Ji Hyun Lee, M.D.^1^, Young Cheol Yoon, M.D.^1^, Wook Jin, M.D.^2^, Jang Gyu Cha, M.D.^3^, Seonwoo Kim, Ph.D.^4^

^1^ Department of Radiology, Samsung Medical Center, Sungkyunkwan University School of Medicine, Seoul, Korea

^2^ Department of Radiology, Kyung Hee University Hospital at Gangdong, Kyung Hee University School of Medicine, Seoul, Korea

^3^ Department of Radiology, Soonchunhyang University Bucheon Hospital, Bucheon, Korea

^4^ Statistics and Data Center, Research Institute for Future Medicine, Samsung Medical Center, Seoul, Korea

**Corresponding author:** Young Cheol Yoon, M.D.

Department of Radiology, Samsung Medical Center, Sungkyunkwan University School of Medicine

50, Ilwon-Dong, Kangnam-Ku, Seoul, 135-710, Korea

Tel: 82-2-3410-6454

Fax: 82-2-3410-0084

E-mail: youngcheol.yoon@gmail.com

**Manuscript type:** Original Article

**Supplemental Material**

1. Morphology: Infiltration was considered present in lesions with indistinct margins. Lobulation was considered present when two or more projections were noted at the margin.
2. Component: Fat was considered present when high signal intensity similar to that of subcutaneous fat on T_1_-weighted imaging and drop in signal on fat-suppressed images were noted without post-contrast enhancement. Fibrosis was defined as low T_1_- and T_2_-weighted signal areas with contrast enhancement. Necrosis was deemed to be present if a fluid-like signal with an irregular margin was observed with no necrotic fluid contrast enhancement. Hemorrhage was considered present when foci with high T_1_-weighted signals did not show signal drop on fat-suppressed images ^1^. Septation was considered present if linear strands traversed the lesion with low T_2_-weighted signal intensity ^2^. The target sign was deemed to be present when a central area of low signal intensity on T_2_-weighted imaging was surrounded by high signal intensity ^3^.
3. Heterogeneity: T_1_ and T_2_ heterogeneity were graded using a four-point scale based on the lesion’s volumetric heterogeneous signal percentage, excluding thin internal septa (0: none, 1: 0–10%, 2: 10–30%, and 3: 30–50%).
4. Perilesional findings: Edema was defined as infiltrative areas of high T_2_ signal surrounding a lesion with low-to-intermediate T_1_ signal that did not modify perilesional anatomical structural architecture. The split fat sign was considered present if a peripheral fat rim surrounding the tumor was noted ^4,5^. The tail sign was considered present when linear enhancement along the aponeurosis extended from tumor margins ^6,7^.
5. Others: Deep location involvement was considered present if the lesion was located deep with respect to the superficial investing fascia or when a superficial tumor crossed the fascia. Neurovascular bundle invasion was deemed to be present when contact between the tumors and vascular or neural circumference exceeded 180° ^8^. Bone invasion was defined as cortical and medullary signal intensity changes and cortical destruction ^9^.

**References**

1. Moulton, J. S. *et al.* MR imaging of soft-tissue masses: diagnostic efficacy and value of distinguishing between benign and malignant lesions. *AJR Am J Roentgenol* **164**, 1191-1199, doi: 10.2214/ajr.164.5.7717231 (1995).

2. Hermann, G., Abdelwahab, I. F., Miller, T. T., Klein, M. J. & Lewis, M. M. Tumour and tumour-like conditions of the soft tissue: magnetic resonance imaging features differentiating benign from malignant masses. *Br J Radiol* **65**, 14-20, doi: 10.1259/0007-1285-65-769-14 (1992).

3. Banks, K. P. The target sign: extremity. *Radiology* **234**, 899-900, doi: 10.1148/radiol.2343030946 (2005).

4. Zhang, Z., Deng, L., Ding, L. & Meng, Q. MR imaging differentiation of malignant soft tissue tumors from peripheral schwannomas with large size and heterogeneous signal intensity. *Eur J Radiol* **84**, 940-946, doi: 10.1016/j.ejrad.2015.02.003 (2015).

5. Walker, E. A., Song, A. J. & Murphey, M. D. Magnetic resonance imaging of soft-tissue masses. *Semin Roentgenol* **45**, 277-297, doi: 10.1053/j.ro.2009.12.004 (2010).

6. Crombe, A. *et al.* Soft tissue masses with myxoid stroma: Can conventional magnetic resonance imaging differentiate benign from malignant tumors? *Eur J Radiol* **85**, 1875-1882, doi: 10.1016/j.ejrad.2016.08.015 (2016).

7. Lefkowitz, R. A. *et al.* Myxofibrosarcoma: prevalence and diagnostic value of the "tail sign" on magnetic resonance imaging. *Skeletal Radiol* **42**, 809-818, doi: 10.1007/s00256-012-1563-6 (2013).

8. Holzapfel, K. *et al.* Local Staging of Soft-Tissue Sarcoma: Emphasis on Assessment of Neurovascular Encasement-Value of MR Imaging in 174 Confirmed Cases. *Radiology* **275**, 501-509, doi: 10.1148/radiol.14140510 (2015).

9. Elias, D. A. *et al.* Osseous invasion by soft-tissue sarcoma: assessment with MR imaging. *Radiology* **229**, 145-152, doi: 10.1148/radiol.2291020377 (2003).
